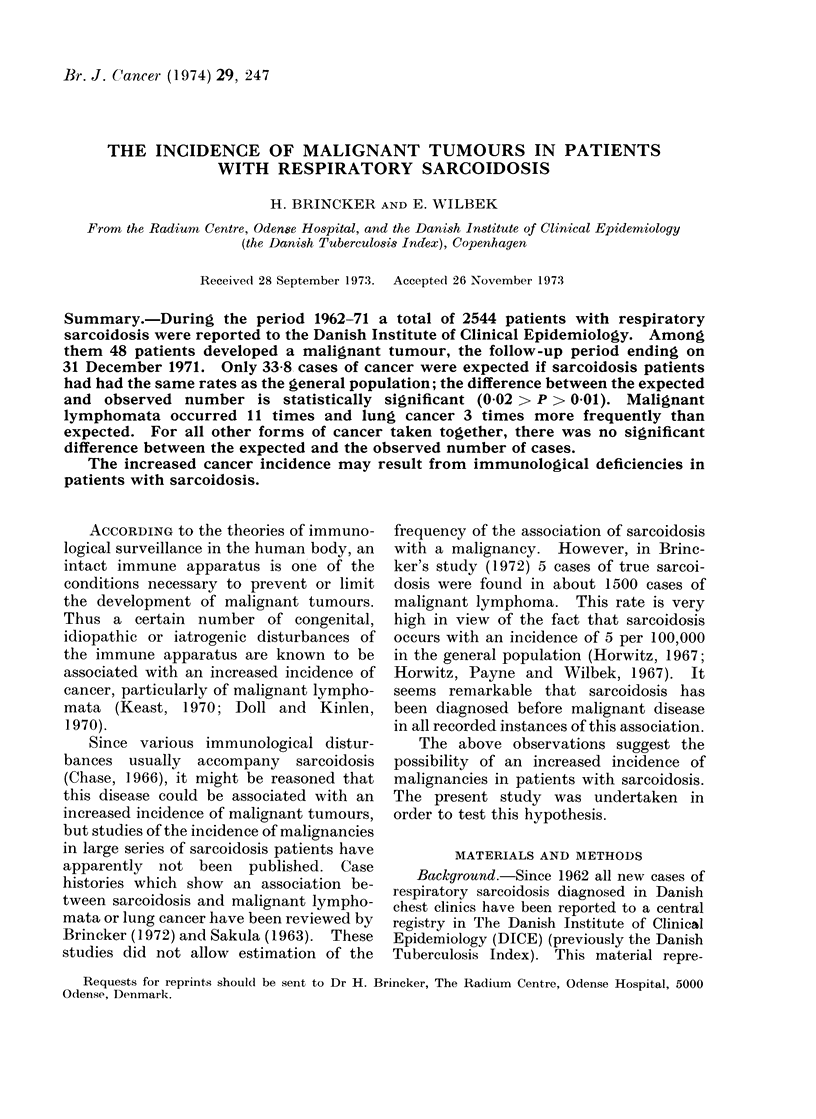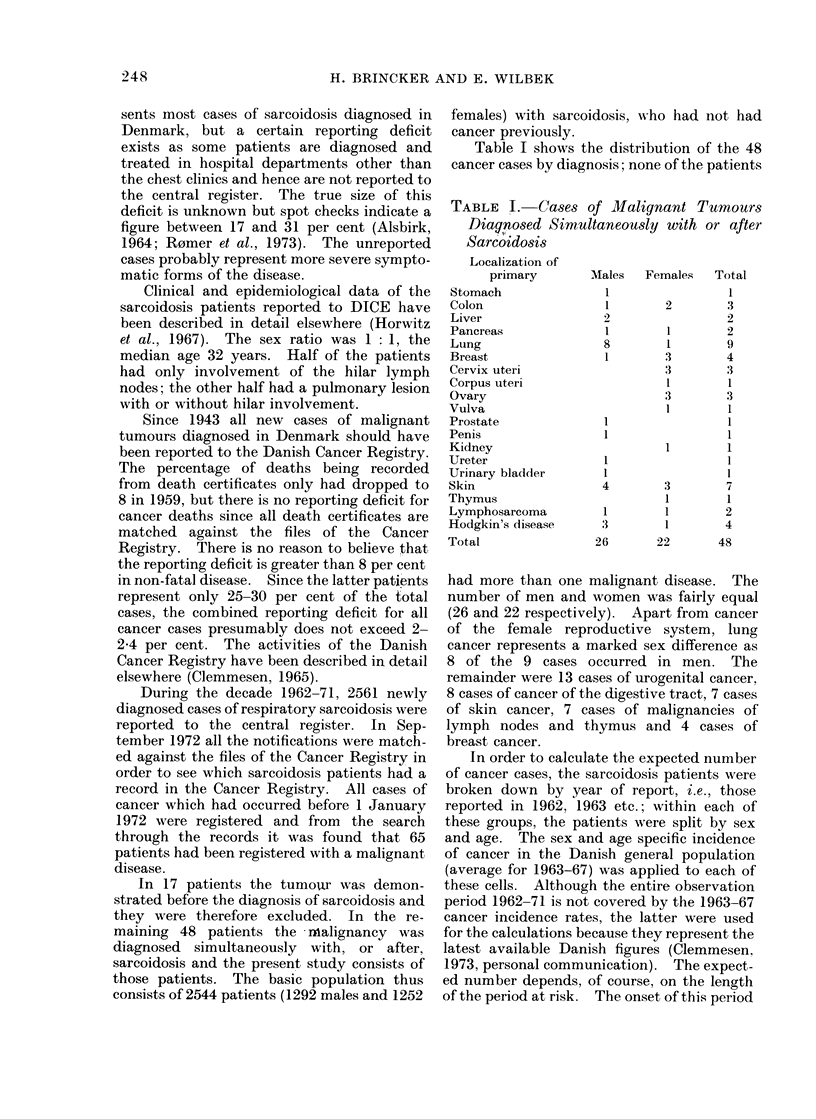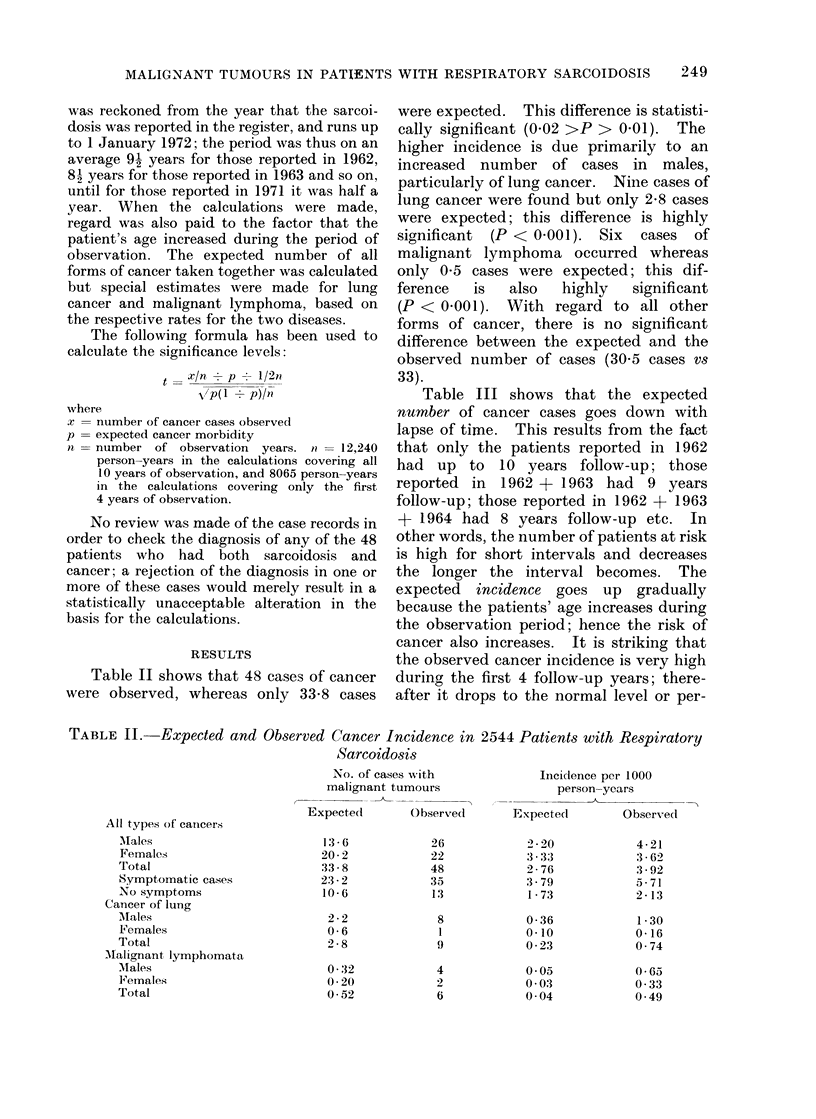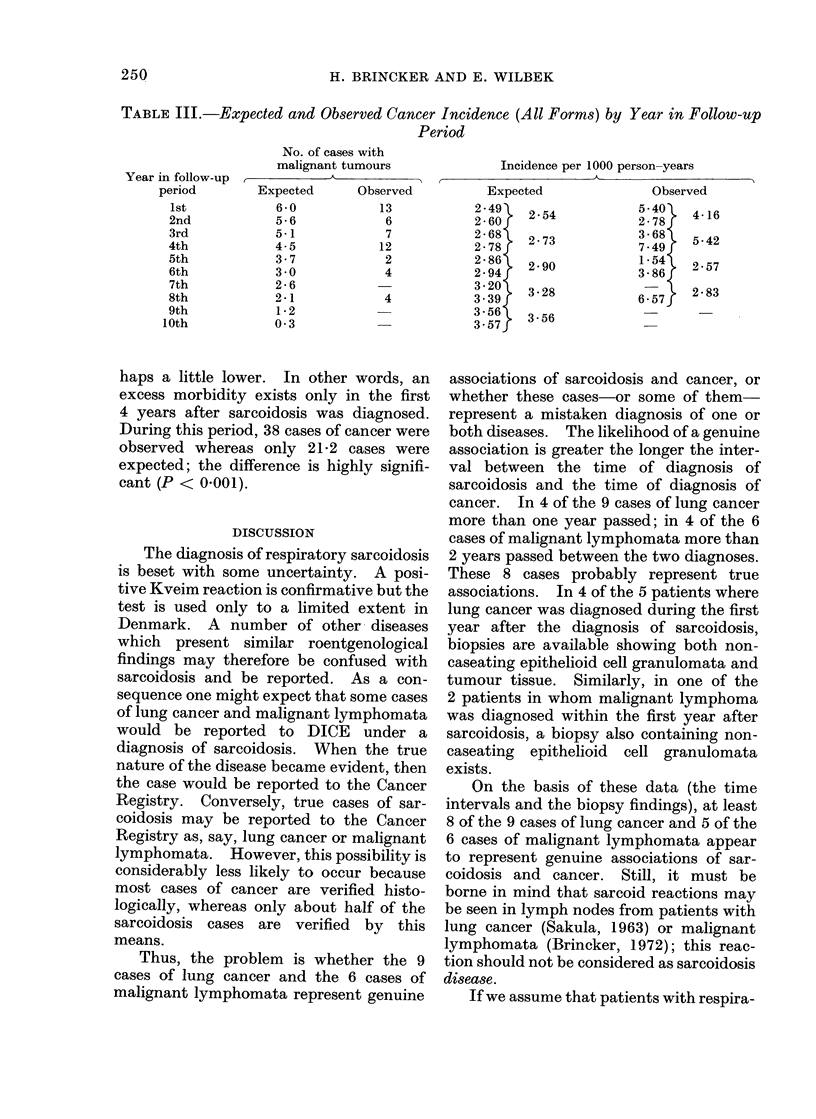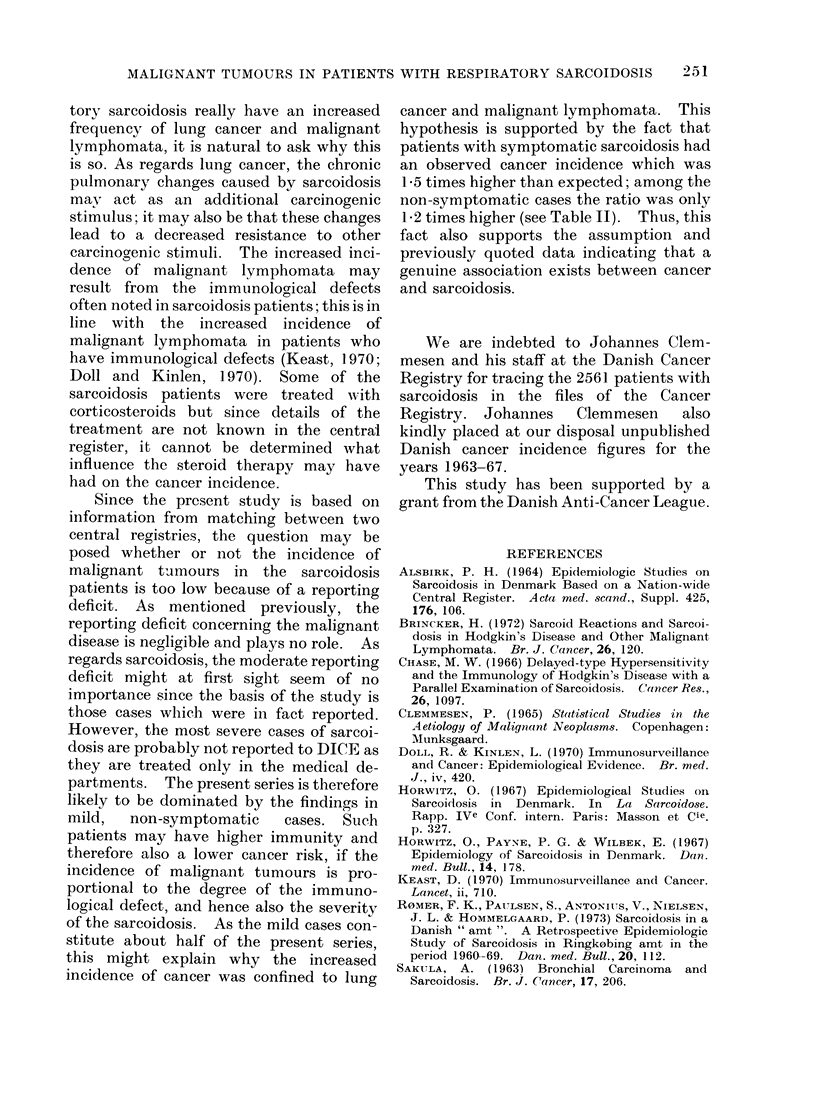# The Incidence of Malignant Tumours in Patients with Respiratory Sarcoidosis

**DOI:** 10.1038/bjc.1974.64

**Published:** 1974-03

**Authors:** H. Brincker, E. Wilbek

## Abstract

During the period 1962-71 a total of 2544 patients with respiratory sarcoidosis were reported to the Danish Institute of Clinical Epidemiology. Among them 48 patients developed a malignant tumour, the follow-up period ending on 31 December 1971. Only 33·8 cases of cancer were expected if sarcoidosis patients had had the same rates as the general population; the difference between the expected and observed number is statistically significant (0·02 > *P* > 0·01). Malignant lymphomata occurred 11 times and lung cancer 3 times more frequently than expected. For all other forms of cancer taken together, there was no significant difference between the expected and the observed number of cases.

The increased cancer incidence may result from immunological deficiencies in patients with sarcoidosis.


*Br. J. Cancer* (1974) **29**, 247

### THE INCIDENCE OF MALIGNANT TUMOURS IN PATIENTS WITH RESPIRATORY SARCOIDOSIS

H. BRINCKER AND E. WILBEK

*From the Radium Centre, Odense Hospital, and the Danish Institute of Clinical Epidemiology (the Danish Tuberculosis Index), Copenhagen*

Received 28 September 1973. Accepted 26 November 1973

**Summary.—During the period 1962–71 a total of 2544 patients with respiratory sarcoidosis were reported to the Danish Institute of Clinical Epidemiology. Among them 48 patients developed a malignant tumour, the follow-up period ending on 31 December 1971. Only 33·8 cases of cancer were expected if sarcoidosis patients had had the same rates as the general population; the difference between the expected and observed number is statistically significant ($0{\cdot}02 > P > 0{\cdot}01$). Malignant lymphomata occurred 11 times and lung cancer 3 times more frequently than expected. For all other forms of cancer taken together, there was no significant difference between the expected and the observed number of cases.**

**The increased cancer incidence may result from immunological deficiencies in patients with sarcoidosis.**

According to the theories of immunological surveillance in the human body, an intact immune apparatus is one of the conditions necessary to prevent or limit the development of malignant tumours. Thus a certain number of congenital, idiopathic or iatrogenic disturbances of the immune apparatus are known to be associated with an increased incidence of cancer, particularly of malignant lymphomata (Keast, 1970; Doll and Kinlen, 1970).

Since various immunological disturbances usually accompany sarcoidosis (Chase, 1966), it might be reasoned that this disease could be associated with an increased incidence of malignant tumours, but studies of the incidence of malignancies in large series of sarcoidosis patients have apparently not been published. Case histories which show an association between sarcoidosis and malignant lymphomata or lung cancer have been reviewed by Brincker (1972) and Sakula (1963). These studies did not allow estimation of the frequency of the association of sarcoidosis with a malignancy. However, in Brincker's study (1972) 5 cases of true sarcoidosis were found in about 1500 cases of malignant lymphoma. This rate is very high in view of the fact that sarcoidosis occurs with an incidence of 5 per 100,000 in the general population (Horwitz, 1967; Horwitz, Payne and Wilbek, 1967). It seems remarkable that sarcoidosis has been diagnosed before malignant disease in all recorded instances of this association.

The above observations suggest the possibility of an increased incidence of malignancies in patients with sarcoidosis. The present study was undertaken in order to test this hypothesis.

### MATERIALS AND METHODS

*Background.*—Since 1962 all new cases of respiratory sarcoidosis diagnosed in Danish chest clinics have been reported to a central registry in The Danish Institute of Clinical Epidemiology (DICE) (previously the Danish Tuberculosis Index). This material repre-

Requests for reprints should be sent to Dr H. Brincker, The Radium Centre, Odense Hospital, 5000 Odense, Denmark.

sents most cases of sarcoidosis diagnosed in Denmark, but a certain reporting deficit exists as some patients are diagnosed and treated in hospital departments other than the chest clinics and hence are not reported to the central register. The true size of this deficit is unknown but spot checks indicate a figure between 17 and 31 per cent (Alsbirk, 1964; Rømer *et al.*, 1973). The unreported cases probably represent more severe symptomatic forms of the disease.

Clinical and epidemiological data of the sarcoidosis patients reported to DICE have been described in detail elsewhere (Horwitz *et al.*, 1967). The sex ratio was 1 : 1, the median age 32 years. Half of the patients had only involvement of the hilar lymph nodes; the other half had a pulmonary lesion with or without hilar involvement.

Since 1943 all new cases of malignant tumours diagnosed in Denmark should have been reported to the Danish Cancer Registry. The percentage of deaths being recorded from death certificates only had dropped to 8 in 1959, but there is no reporting deficit for cancer deaths since all death certificates are matched against the files of the Cancer Registry. There is no reason to believe that the reporting deficit is greater than 8 per cent in non-fatal disease. Since the latter patients represent only 25–30 per cent of the total cases, the combined reporting deficit for all cancer cases presumably does not exceed 2–2·4 per cent. The activities of the Danish Cancer Registry have been described in detail elsewhere (Clemmesen, 1965).

During the decade 1962–71, 2561 newly diagnosed cases of respiratory sarcoidosis were reported to the central register. In September 1972 all the notifications were matched against the files of the Cancer Registry in order to see which sarcoidosis patients had a record in the Cancer Registry. All cases of cancer which had occurred before 1 January 1972 were registered and from the search through the records it was found that 65 patients had been registered with a malignant disease.

In 17 patients the tumour was demonstrated before the diagnosis of sarcoidosis and they were therefore excluded. In the remaining 48 patients the malignancy was diagnosed simultaneously with, or after, sarcoidosis and the present study consists of those patients. The basic population thus consists of 2544 patients (1292 males and 1252 females) with sarcoidosis, who had not had cancer previously.

Table I shows the distribution of the 48 cancer cases by diagnosis; none of the patients

TABLE I.—*Cases of Malignant Tumours Diagnosed Simultaneously with or after Sarcoidosis*

| Localization of primary | Males | Females | Total |
|---|---|---|---|
| Stomach | 1 | | 1 |
| Colon | 1 | 2 | 3 |
| Liver | 2 | | 2 |
| Pancreas | 1 | 1 | 2 |
| Lung | 8 | 1 | 9 |
| Breast | 1 | 3 | 4 |
| Cervix uteri | | 3 | 3 |
| Corpus uteri | | 1 | 1 |
| Ovary | | 3 | 3 |
| Vulva | | 1 | 1 |
| Prostate | 1 | | 1 |
| Penis | 1 | | 1 |
| Kidney | | 1 | 1 |
| Ureter | 1 | | 1 |
| Urinary bladder | 1 | | 1 |
| Skin | 4 | 3 | 7 |
| Thymus | | 1 | 1 |
| Lymphosarcoma | 1 | 1 | 2 |
| Hodgkin's disease | 3 | 1 | 4 |
| Total | 26 | 22 | 48 |

had more than one malignant disease. The number of men and women was fairly equal (26 and 22 respectively). Apart from cancer of the female reproductive system, lung cancer represents a marked sex difference as 8 of the 9 cases occurred in men. The remainder were 13 cases of urogenital cancer, 8 cases of cancer of the digestive tract, 7 cases of skin cancer, 7 cases of malignancies of lymph nodes and thymus and 4 cases of breast cancer.

In order to calculate the expected number of cancer cases, the sarcoidosis patients were broken down by year of report, *i.e.*, those reported in 1962, 1963 etc.; within each of these groups, the patients were split by sex and age. The sex and age specific incidence of cancer in the Danish general population (average for 1963–67) was applied to each of these cells. Although the entire observation period 1962–71 is not covered by the 1963–67 cancer incidence rates, the latter were used for the calculations because they represent the latest available Danish figures (Clemmesen, 1973, personal communication). The expected number depends, of course, on the length of the period at risk. The onset of this period was reckoned from the year that the sarcoidosis was reported in the register, and runs up to 1 January 1972; the period was thus on an average $9\frac{1}{2}$ years for those reported in 1962, $8\frac{1}{2}$ years for those reported in 1963 and so on, until for those reported in 1971 it was half a year. When the calculations were made, regard was also paid to the factor that the patient's age increased during the period of observation. The expected number of all forms of cancer taken together was calculated but special estimates were made for lung cancer and malignant lymphoma, based on the respective rates for the two diseases.

The following formula has been used to calculate the significance levels:

$$t = \frac{x/n \div p \div 1/2n}{\sqrt{p(1 \div p)/n}}$$

where
$x$ = number of cancer cases observed
$p$ = expected cancer morbidity
$n$ = number of observation years. $n$ = 12,240 person–years in the calculations covering all 10 years of observation, and 8065 person–years in the calculations covering only the first 4 years of observation.

No review was made of the case records in order to check the diagnosis of any of the 48 patients who had both sarcoidosis and cancer; a rejection of the diagnosis in one or more of these cases would merely result in a statistically unacceptable alteration in the basis for the calculations.

### RESULTS

Table II shows that 48 cases of cancer were observed, whereas only 33·8 cases were expected. This difference is statistically significant ($0{\cdot}02 > P > 0{\cdot}01$). The higher incidence is due primarily to an increased number of cases in males, particularly of lung cancer. Nine cases of lung cancer were found but only 2·8 cases were expected; this difference is highly significant ($P < 0{\cdot}001$). Six cases of malignant lymphoma occurred whereas only 0·5 cases were expected; this difference is also highly significant ($P < 0{\cdot}001$). With regard to all other forms of cancer, there is no significant difference between the expected and the observed number of cases (30·5 cases *vs* 33).

Table III shows that the expected *number* of cancer cases goes down with lapse of time. This results from the fact that only the patients reported in 1962 had up to 10 years follow-up; those reported in 1962 + 1963 had 9 years follow-up; those reported in 1962 + 1963 + 1964 had 8 years follow-up etc. In other words, the number of patients at risk is high for short intervals and decreases the longer the interval becomes. The expected *incidence* goes up gradually because the patients' age increases during the observation period; hence the risk of cancer also increases. It is striking that the observed cancer incidence is very high during the first 4 follow-up years; thereafter it drops to the normal level or per-

TABLE II.—*Expected and Observed Cancer Incidence in 2544 Patients with Respiratory Sarcoidosis*

| | No. of cases with malignant tumours | | Incidence per 1000 person–years | |
|---|---|---|---|---|
| | Expected | Observed | Expected | Observed |
| All types of cancers | | | | |
| Males | 13·6 | 26 | 2·20 | 4·21 |
| Females | 20·2 | 22 | 3·33 | 3·62 |
| Total | 33·8 | 48 | 2·76 | 3·92 |
| Symptomatic cases | 23·2 | 35 | 3·79 | 5·71 |
| No symptoms | 10·6 | 13 | 1·73 | 2·13 |
| Cancer of lung | | | | |
| Males | 2·2 | 8 | 0·36 | 1·30 |
| Females | 0·6 | 1 | 0·10 | 0·16 |
| Total | 2·8 | 9 | 0·23 | 0·74 |
| Malignant lymphomata | | | | |
| Males | 0·32 | 4 | 0·05 | 0·65 |
| Females | 0·20 | 2 | 0·03 | 0·33 |
| Total | 0·52 | 6 | 0·04 | 0·49 |

TABLE III.—*Expected and Observed Cancer Incidence (All Forms) by Year in Follow-up Period*

| Year in follow-up period | No. of cases with malignant tumours | | Incidence per 1000 person–years | | | |
|---|---|---|---|---|---|---|
| | Expected | Observed | Expected | | Observed | |
| 1st | 6·0 | 13 | 2·49 | 2·54 | 5·40 | 4·16 |
| 2nd | 5·6 | 6 | 2·60 | | 2·78 | |
| 3rd | 5·1 | 7 | 2·68 | 2·73 | 3·68 | 5·42 |
| 4th | 4·5 | 12 | 2·78 | | 7·49 | |
| 5th | 3·7 | 2 | 2·86 | 2·90 | 1·54 | 2·57 |
| 6th | 3·0 | 4 | 2·94 | | 3·86 | |
| 7th | 2·6 | — | 3·20 | 3·28 | — | 2·83 |
| 8th | 2·1 | 4 | 3·39 | | 6·57 | |
| 9th | 1·2 | — | 3·56 | 3·56 | — | — |
| 10th | 0·3 | — | 3·57 | | — | |

haps a little lower. In other words, an excess morbidity exists only in the first 4 years after sarcoidosis was diagnosed. During this period, 38 cases of cancer were observed whereas only 21·2 cases were expected; the difference is highly significant ($P < 0{\cdot}001$).

### DISCUSSION

The diagnosis of respiratory sarcoidosis is beset with some uncertainty. A positive Kveim reaction is confirmative but the test is used only to a limited extent in Denmark. A number of other diseases which present similar roentgenological findings may therefore be confused with sarcoidosis and be reported. As a consequence one might expect that some cases of lung cancer and malignant lymphomata would be reported to DICE under a diagnosis of sarcoidosis. When the true nature of the disease became evident, then the case would be reported to the Cancer Registry. Conversely, true cases of sarcoidosis may be reported to the Cancer Registry as, say, lung cancer or malignant lymphomata. However, this possibility is considerably less likely to occur because most cases of cancer are verified histologically, whereas only about half of the sarcoidosis cases are verified by this means.

Thus, the problem is whether the 9 cases of lung cancer and the 6 cases of malignant lymphomata represent genuine associations of sarcoidosis and cancer, or whether these cases—or some of them—represent a mistaken diagnosis of one or both diseases. The likelihood of a genuine association is greater the longer the interval between the time of diagnosis of sarcoidosis and the time of diagnosis of cancer. In 4 of the 9 cases of lung cancer more than one year passed; in 4 of the 6 cases of malignant lymphomata more than 2 years passed between the two diagnoses. These 8 cases probably represent true associations. In 4 of the 5 patients where lung cancer was diagnosed during the first year after the diagnosis of sarcoidosis, biopsies are available showing both non-caseating epithelioid cell granulomata and tumour tissue. Similarly, in one of the 2 patients in whom malignant lymphoma was diagnosed within the first year after sarcoidosis, a biopsy also containing non-caseating epithelioid cell granulomata exists.

On the basis of these data (the time intervals and the biopsy findings), at least 8 of the 9 cases of lung cancer and 5 of the 6 cases of malignant lymphomata appear to represent genuine associations of sarcoidosis and cancer. Still, it must be borne in mind that sarcoid reactions may be seen in lymph nodes from patients with lung cancer (Sakula, 1963) or malignant lymphomata (Brincker, 1972); this reaction should not be considered as sarcoidosis *disease*.

If we assume that patients with respiratory sarcoidosis really have an increased frequency of lung cancer and malignant lymphomata, it is natural to ask why this is so. As regards lung cancer, the chronic pulmonary changes caused by sarcoidosis may act as an additional carcinogenic stimulus; it may also be that these changes lead to a decreased resistance to other carcinogenic stimuli. The increased incidence of malignant lymphomata may result from the immunological defects often noted in sarcoidosis patients; this is in line with the increased incidence of malignant lymphomata in patients who have immunological defects (Keast, 1970; Doll and Kinlen, 1970). Some of the sarcoidosis patients were treated with corticosteroids but since details of the treatment are not known in the central register, it cannot be determined what influence the steroid therapy may have had on the cancer incidence.

Since the present study is based on information from matching between two central registries, the question may be posed whether or not the incidence of malignant tumours in the sarcoidosis patients is too low because of a reporting deficit. As mentioned previously, the reporting deficit concerning the malignant disease is negligible and plays no role. As regards sarcoidosis, the moderate reporting deficit might at first sight seem of no importance since the basis of the study is those cases which were in fact reported. However, the most severe cases of sarcoidosis are probably not reported to DICE as they are treated only in the medical departments. The present series is therefore likely to be dominated by the findings in mild, non-symptomatic cases. Such patients may have higher immunity and therefore also a lower cancer risk, if the incidence of malignant tumours is proportional to the degree of the immunological defect, and hence also the severity of the sarcoidosis. As the mild cases constitute about half of the present series, this might explain why the increased incidence of cancer was confined to lung cancer and malignant lymphomata. This hypothesis is supported by the fact that patients with symptomatic sarcoidosis had an observed cancer incidence which was 1·5 times higher than expected; among the non-symptomatic cases the ratio was only 1·2 times higher (see Table II). Thus, this fact also supports the assumption and previously quoted data indicating that a genuine association exists between cancer and sarcoidosis.

We are indebted to Johannes Clemmesen and his staff at the Danish Cancer Registry for tracing the 2561 patients with sarcoidosis in the files of the Cancer Registry. Johannes Clemmesen also kindly placed at our disposal unpublished Danish cancer incidence figures for the years 1963–67.

This study has been supported by a grant from the Danish Anti-Cancer League.

REFERENCES

ALSBIRK, P. H. (1964) Epidemiologic Studies on Sarcoidosis in Denmark Based on a Nation-wide Central Register. *Acta med. scand.*, Suppl. 425, **176**, 106.

BRINCKER, H. (1972) Sarcoid Reactions and Sarcoidosis in Hodgkin's Disease and Other Malignant Lymphomata. *Br. J. Cancer*, **26**, 120.

CHASE, M. W. (1966) Delayed-type Hypersensitivity and the Immunology of Hodgkin's Disease with a Parallel Examination of Sarcoidosis. *Cancer Res.*, **26**, 1097.

CLEMMESEN, P. (1965) *Statistical Studies in the Aetiology of Malignant Neoplasms*. Copenhagen: Munksgaard.

DOLL, R. & KINLEN, L. (1970) Immunosurveillance and Cancer: Epidemiological Evidence. *Br. med. J.*, iv, 420.

HORWITZ, O. (1967) Epidemiological Studies on Sarcoidosis in Denmark. In *La Sarcoidose*. Rapp. IVe Conf. intern. Paris: Masson et Cie. p. 327.

HORWITZ, O., PAYNE, P. G. & WILBEK, E. (1967) Epidemiology of Sarcoidosis in Denmark. *Dan. med. Bull.*, **14**, 178.

KEAST, D. (1970) Immunosurveillance and Cancer. *Lancet*, ii, 710.

RØMER, F. K., PAULSEN, S., ANTONIUS, V., NIELSEN, J. L. & HOMMELGAARD, P. (1973) Sarcoidosis in a Danish " amt ". A Retrospective Epidemiologic Study of Sarcoidosis in Ringkøbing amt in the period 1960–69. *Dan. med. Bull.*, **20**, 112.

SAKULA, A. (1963) Bronchial Carcinoma and Sarcoidosis. *Br. J. Cancer*, **17**, 206.